# Rapid Development of Small Rodent Animal Models for Infectious Disease Research Through Vectorized Receptor Molecule Expression

**DOI:** 10.3390/v16111794

**Published:** 2024-11-19

**Authors:** Melanie M. Goens, Erin L. Howard, Bryce M. Warner, Leonardo Susta, Sarah K. Wootton

**Affiliations:** 1 Department of Pathobiology, Ontario Veterinary College, University of Guelph, Guelph, ON N1G 2W1, Canada; 2 Vaccine and Infectious Disease Organization, University of Saskatchewan, 120 Veterinary Rd, Saskatoon, SK S7N 5E3, Canada; brw566@usask.ca; 3 Department of Biochemistry, Microbiology, and Immunology, University of Saskatchewan, 107 Wiggins Rd, Saskatoon, SK S7N 5E5, Canada

**Keywords:** adeno-associated virus vector, animal models, zoonotic infections, pandemic potential, viral countermeasures, prophylactic and therapeutic strategies

## Abstract

The emergence and re-emergence of pathogens with pandemic potential has been a persistent issue throughout history. Recent decades have seen significant outbreaks of zoonotic viruses from members of the *Coronaviridae*, *Filoviridae*, *Paramyxoviridae*, *Flaviviridae*, and *Togaviridae* families, resulting in widespread infections. The continual emergence of zoonotic viral pathogens and associated infections highlights the need for prevention strategies and effective treatments. Central to this effort is the availability of suitable animal models, which are essential for understanding pathogenesis and assessing transmission dynamics. These animals are also critical for evaluating the safety and efficacy of novel vaccines or therapeutics and are essential in facilitating regulatory approval of new products. Rapid development of animal models is an integral aspect of pandemic response and preparedness; however, their establishment is fraught by several rate-limiting steps, including selection of a suitable species, the logistical challenges associated with sharing and disseminating transgenic animals (e.g., the time-intensive nature of breeding and maintaining colonies), the availability of technical expertise, as well as ethical and regulatory approvals. A method for the rapid development of relevant animal models that has recently gained traction, in large part due to the COVID-19 pandemic, is the use of gene therapy vectors to express human viral receptors in readily accessible laboratory animals to enable virus infection and development of clinical disease. These models can be developed rapidly on any genetic background, making mechanistic studies and accelerated evaluation of novel countermeasures possible. In this review, we will discuss important considerations for the effective development of animal models using viral vector approaches and review the current vector-based animal models for studying viral pathogenesis and evaluating prophylactic and therapeutic strategies, with an emphasis on models of SARS-CoV-2 infection based on the vectorized expression of human angiotensin-converting enzyme 2.

## 1. Introduction

With the continued emergence of zoonotic viral pathogens, many with known or possible pandemic potential, there is great urgency to develop effective treatment and prevention strategies. For this to happen, scientists need access to suitable animal models that mimic natural infection and recapitulate important aspects of human disease in order to understand the pathogenesis of the virus and its transmission dynamics, to identify correlates of protection, and ultimately, to evaluate novel vaccines and therapeutics [1].

## 2. Conventional Methods for Development of Mouse Models to Study Human Virus Infections

Replication of human viruses in non-native hosts, such as laboratory mice, can be impeded at several stages in the virus life cycle. The first step in the virus life cycle is attachment to a receptor on the cell surface and delivery of the viral genome and associated proteins into the cytoplasm [2]. There are several examples of a human viruses, including poliovirus (CD155 [3]), human rhinovirus (ICAM1 [4]), hepatitis C virus (HCV; CD81 and occludin [5,6,7]), SARS-CoV (ACE2 [8]), and MERS-CoV (hDPP4 [9]), among others, that have drastically decreased receptor affinity to the mouse orthologs of human host cell receptors due to amino acid differences, ultimately preventing entry into the cell. Moreover, in some cases, the primary attachment receptor is insufficient to mediate infection alone, and a co-receptor, as in the case of HIV, is necessary for productive virus entry [10]. Lastly, even if entry is achieved, the cells may not be permissive due to differences in the cellular machinery of the new host, which might not fully support viral replication due to species-specific variations in intracellular processes and pattern-recognition receptors [10]. Nonetheless, in many instances, mice become susceptible to human viruses when they artificially express the proper human receptors, making permissivity considerations less stringent [11].

Microinjection of recombinant DNA (i.e., transgene) into the pronucleus of a fertilized egg (i.e., pronuclear microinjection) is the most widely used method for creating transgenic mammals [12], even as several new techniques have been developed [13]. Importantly, this technique was used to produce a mouse model for severe acute respiratory syndrome (SARS), caused by the SARS coronavirus (SARS-CoV). This was accomplished by introducing the human angiotensin-converting enzyme 2 (hACE2) coding sequence, under the control of the human cytokeratin 18 (K18) promoter, into a C57BL/6 background, allowing for productive infection with SARS-CoV and the development of a rapidly fatal respiratory and neurological disease [8]. Microinjection, which originated almost 40 years ago, has remained valuable in the gene-editing field as it is able to deliver multiple components into embryos, including DNA, zinc fingers, and CRISPR-Cas9, which can be used extensively to modify the mouse genome. The CRISPR-Cas9 approach was employed to generate a mouse model for MERS-CoV, whereby gene editing was used to modify the murine dipeptidyl peptidase 4 receptor (DPP4) to encode two amino acids (positions 288 and 330) to match the human ortholog sequence, making mice susceptible to MERS-CoV infection and replication [14].

Another approach to studying human viruses in laboratory animals is to species-adapt the virus to the model host through serial passaging. There are several examples of human viruses that have been “mouse-adapted”, including Ebola virus (MA-EBOV), Marburg virus (MA-MARV), and SARS-CoV-2 (MA-SARS-CoV-2), to name a few [15,16,17,18]. In some cases, however, human viruses need to be serially passaged in transgenic mice that express the human receptor, as was the case for MERS-CoV [19]. While species-adapted viruses are important tools for studying virus biology and countermeasures, the process is time-consuming, with the major drawback being that any novel therapeutic would not be tested using the wild-type virus. Moreover, serial passaging of mammalian zoonotic pathogens for adaptation purposes may fall under gain-of-function regulatory frameworks, potentially restricting its application and underscoring the advantage of utilizing viral vector approaches.

**Figure 1:**
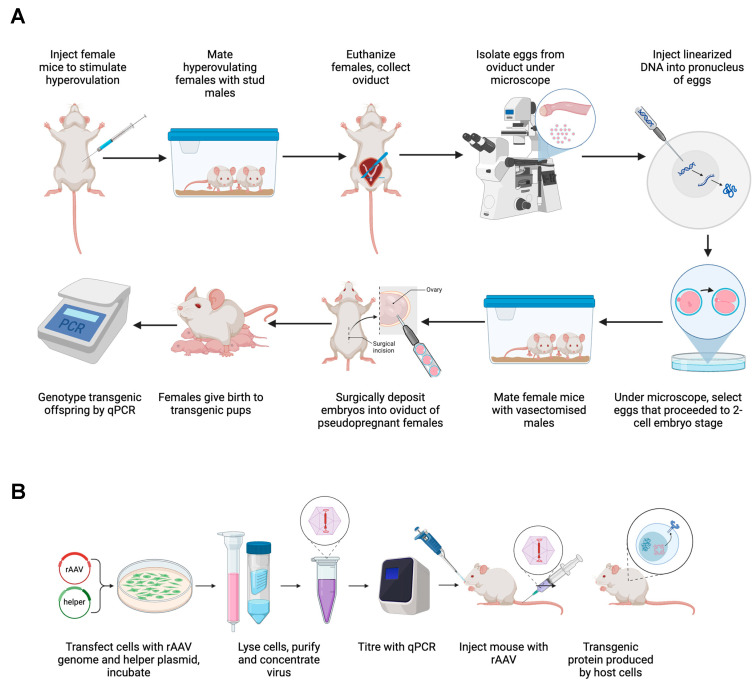
Schematic of the workflow involved in (**A**) creating transgenic mice using pronuclear microinjection into fertilized eggs [20] or (**B**) viral vector-mediated expression of viral entry receptors to develop mouse models susceptible to human viral infections. Created using BioRender.

Creating transgenic mice or mouse-adapted viruses is both technically demanding and time-consuming, requiring specialized expertise and equipment [12,20]. In the event of a pandemic, alternative and complementary methods for the rapid development of translational models, such as vectorized expression of receptor molecules in non-germinally transgenic animals, may need to be mobilized [21] (Figure 1).

## 3. Viral-Vectored Receptor Expression as an Alternative Method for Infectious Disease Mouse Model Development

Viral vectors have been extensively utilized for in vivo gene delivery, not only for therapeutic applications but also for the development of mouse models of viral infections. Given their remarkable flexibility, researchers can, in a time- and cost-effective manner, use viral vectors to express transgenes, including virus attachment proteins, at different doses, targeting diverse tissues across different ages and species, to model and study the infection of human viruses in laboratory animals. Compared with generating germinally transgenic mice, using viral vectors to create mouse models of infection is both quicker and more versatile. As with all model systems, there are both advantages and disadvantages that must be considered, and these are briefly described in Table 1.

For the development of mouse models susceptible to human viral infections, adeno-associated virus (AAV) and adenovirus (AdV) gene therapy vectors are the preferred gene delivery vehicles [22].

### 3.1. Adeno-Associated Virus Vectors for In Vivo Gene Delivery

Adeno-associated virus (AAV) is a non-enveloped helper-dependent virus belonging to the *Parvoviridae* family. AAV is currently the leading platform for in vivo gene transfer, with eight approved therapies on the market: Glybera [23], Luxturna [24], Zolgensma [25], Hemgenix [26], Elevidys [27], Roctavian [28], Upstaza [29], and Beqvez [30]. AAV comprises a single-stranded DNA genome approximately 4.7 kb in length encoding a rep and cap gene flanked by two inverted terminal repeats (ITRs) that form hairpin-like structures at the 5′ and 3′ ends of the genome. (Rep refers to the replication gene, and Cap refers to the capsid gene required for viral replication and virion packaging, respectively.) The rep and cap genes are removed from AAV vectors to make space for the transgene and necessary regulatory elements, and these proteins are provided in trans during manufacturing, typically in HEK 293 cells (either adherent or suspension), to allow for genome replication and packaging [31]. AAV mediates sustained transgene expression from its episomal genome [32] and is known to be minimally immunogenic, making this vector highly attractive for in vivo gene delivery [33,34]. Currently, there are at least 13 AAV serotypes with varying tissue tropism and transducing properties [35]. AAV has been used in hundreds of clinical trials targeting the brain, liver, eye, lung, muscle, and other tissues, making it one of the most well-studied in vivo gene delivery systems [36].

### 3.2. Adenovirus Vectors for In Vivo Gene Delivery

Adenoviruses (AdVs) are non-enveloped double-stranded DNA viruses that infect a broad range of vertebrate species and are known to cause mild respiratory and gastrointestinal infections. Most infections with adenoviruses in humans occur in the upper respiratory tract, causing symptoms such as the common cold and tonsillitis. Human adenoviruses (HAds) are classified into 50 serotypes divided into 6 subgroups (A to F) based on cross-neutralization by antibodies. AdV serotype 5 (Ad5) has been used as a viral vector for gene transfer due to its highly efficient infectious properties, though its immunogenic nature makes it less safe, especially the first-generation vector design [37,38]. However, third-generation or “gutted” helper-dependent AdV (HD-Ad) vectors are far less immunogenic and mediate long-term transgene expression [39,40].

### 3.3. Comparison of AAV vs. Adenovirus Vectors for In Vivo Receptor Gene Delivery

While AdV vectors are useful for applications requiring high-level, short-term transgene expression, AAV vectors are preferred for achieving longer-term transgene expression due to their lower immunogenicity and more stable genome presence. From the point of view of the kinetics of transgene expression, AdV tends to reach peak transgene expression faster than AAV, thus shortening the lead time between administration of the receptor-expressing vector and virus infection (See Table 2 and Table 3 for a comparison of the time intervals between vector administration and virus challenge). One advantage of AdV over AAV is its packaging capacity; while AAV vectors are generally limited to 4.7 kb, HD-Ad vectors can accommodate between 28 and 38 kb [41]. Despite this, the higher immunogenicity of AdV vectors can cause the development of background inflammation and immune activation, complicating the interpretation of the results of infectious disease studies [42]. In terms of tissue tropism, there are numerous serotype options for AAV vector pseudotyping, many of which have well-defined tropisms, and when combined with tissue-specific promoters, can allow for targeted delivery and expression of viral host receptors. Moreover, a new generation of novel AAV vectors is being developed through the discovery of naturally occurring AAV isolates in different animal species, rational capsid design, capsid shuffling, chimeric capsid construction, and peptide display, as well as directed evolution involving complex capsid libraries [43,44].

Producing HD-Ad vectors, which lack all viral coding sequences, relies on the use of a helper virus for replication and packaging [70]. A significant challenge of HD-Ad vector production is helper virus contamination, and ensuring the separation of HD-Ad from the helper virus requires precise and efficient methods to minimize contamination and ensure purity [70,71]. Production of AAV vectors, on the other hand, is more straightforward and depends on transfection of the AAV genome, packaging, and helper plasmids to produce virus particles, which are then purified using affinity chromatography or ultracentrifugation [72]. Since the production of recombinant AAV vectors does not require the use of a helper virus, no additional purification steps are needed.

Both AAV and AdV vectors have their unique advantages, and the choice between them depends on the specific requirements of the animal model, including the desired duration of gene expression, the target tissue characteristics, and the expertise of the lab.

## 4. Examples of Viral-Vectored Expression for Rapid Development of Mouse Models of Human Virus Infections

### 4.1. MERS-CoV

The Middle East respiratory syndrome (MERS) coronavirus is a betacoronavirus first identified in humans in Saudi Arabia and Jordan in 2012 [73]. Camelids are a known reservoir of MERS-CoV, but other species may act as hosts [74]. Known to cause severe pneumonia in humans, MERS-CoV has a case fatality rate of approximately 36%. Since April 2012, and as of August 2024, a total of 2622 cases of MERS, including 953 deaths, have been reported by health authorities in 27 different countries (World Health Organization, 2024). This zoonotic pathogen can be spread human-to-human, raising the concern of pandemic potential, especially considering that three lethal zoonotic diseases that have emerged in the last 17 years have all been caused by betacoronaviruses [75]. Shortly after the emergence of MERS-CoV, Raj et al. identified dipeptidyl peptidase 4 (DPP4) as a functional receptor for MERS-CoV using an immunoadhesin comprising the S1 domain of the MERS-CoV spike protein fused to the Fc region of human IgG in a pulldown assay [76]. Zhao et al. then went on to investigate whether exogenous expression of human DPP4 (hDPP4) would render mice susceptible to MERS-CoV infection. To do this, the researchers engineered a replication-defective adenovirus to express the hDPP4 receptor (Ad5-hDPP4) in mice [77]. The ability of Ad5-hDPP4 to render mice susceptible to MERS-CoV infection was tested in 6- to 12-week-old and 18- to 22-month-old C57BL/6 and BALB/c mice 5 days post-intranasal administration of 2.5 × 10^8^ PFU Ad5-hDPP4, as the authors showed that any innate responses triggered by the Ad vector had largely dissipated by this time point. The Ad5-hDPP4-sensitized mice showed clinical signs of MERS-CoV infection, with viral replication in lung tissue reaching ∼10^7^ pfu/g lung tissue by 2–3 days post-challenge and evidence of immune cell infiltration and interstitial pneumonia. Using various strains of commercially available knockout mice, the investigators demonstrated that innate (Ad5-hDPP4-transduced IFNAR^−/−^, MAVS^−/−^, and MyD88^−/−^ mice), antibody (Ad5-hDPP4-transduced recombination activating gene 1^−/−^ (RAG1^−/−^) severe combined immunodeficiency (SCID) mice), and T-cell (Ad5-hDPP4-transduced T-cell receptor α^−/−^ (TCRα^−/−^) mice) mediated responses are important for protection against MERS-CoV. To further address the role of IFN signaling in MERS-CoV protection, Ad5-hDPP4-sensitized C57BL/6 mice were pre-treated with poly I:C, IFN-β, or IFN-γ 6 h before MERS-CoV challenge. Poly I:C and IFN-β were found to accelerate virus clearance. The ability to employ vectorized expression of a receptor molecule to rapidly develop a mouse model that supports MERS-CoV infection and allows for detailed analysis of pathogenic mechanisms highlights the many advantages of this approach.

### 4.2. SARS-CoV-2

#### 4.2.1. AAV-Vectorized Expression of hACE2

Severe acute respiratory syndrome coronavirus (SARS-CoV) was first reported in humans in late 2002, and although the outbreak was over in roughly nine months, it infected more than 8000 people, causing 774 deaths in 30 countries [1,78]. Global efforts to study the pathogenesis of SARS-CoV and to evaluate therapies and vaccine candidates were hindered due to the lack of animal models and the fact that it took several months to screen animal species for susceptibility to SARS-CoV. Although SARS-CoV was not highly transmissible and thus did not lead to a pandemic, this experience highlighted the need for the rapid development of animal models in the context of emerging diseases. SARS-CoV-2 was first reported in China in December 2019, and unlike SARS-CoV, it resulted in a worldwide pandemic [79]. SARS-CoV-2 is closely related to SARS-CoV and utilizes the same cell entry receptor, the angiotensin-converting enzyme 2 (ACE2). Despite utilizing the same cellular receptor for virus entry into cells, these two viruses differ from each other in terms of pathogenesis and transmissibility, whereby SARS-CoV-2 induces milder lung lesions, has a lower fatality rate, exhibits less neutralizing antibody generation, and is more transmissible than SARS-CoV [80,81]. Mice are not susceptible to infection with SARS-CoV or SARS-CoV-2 since mouse ACE2 does not serve as a receptor for these viruses [51]. While hACE2 transgenic mice are susceptible to infection and clinical disease, these mice were limited in availability at the beginning of the coronavirus disease 2019 (COVID-19) pandemic and are restricted to a single genetic background [8]. Although other animal models have been shown to be susceptible to authentic SARS-CoV [82] and SARS-CoV-2 [83], such as hamsters, ferrets, and non-human primates (NHPs), these species present challenges due to their relatively high costs, limited availability, housing complexities, and, in certain instances, limited reagent availability relative to that of mice. The first report describing vectored expression of hACE2 as a way to permit SARS-CoV-2 replication in wild-type mice was in late 2020. Israelow et al. administered 1 × 10^11^ vector genomes (vgs) AAV9-CMV-hACE2 vector intratracheally to C57BL/6 mice and challenged them with 1.5 × 10^6^ PFU of ancestral SARS-CoV-2 two weeks later [45]. Lung homogenates from AAV9-hACE2 transduced mice showed a >200-fold increase in SARS-CoV-2 RNA compared with control mice transduced with AAV-GFP or PBS, as well as the presence of infectious virus at 2 and 4 days post-infection. Histopathological analysis of lung tissue showed the presence of mild diffuse peribronchial infiltrates in the AAV9-hACE2 mice, which was negligible in the control mice, as well as evidence of SARS-CoV-2 N protein expression within the alveolar epithelia. The authors went on to demonstrate the power of this vectored receptor expression approach to interrogate the mechanisms of pathogenesis by transducing interferon-α receptor-deficient B6/J mice (IFNAR^−/−^) and IFN regulatory transcription factor 3/7 double-KO mice with AAV9-hACE2. The authors demonstrated that the absence of type I interferon signaling did not affect SARS-CoV-2 replication in vivo. However, it significantly impaired the recruitment of inflammatory cells to the lungs, indicating that type I IFN signaling plays a role in the pathological immune response observed during SARS-CoV-2 infection.

Tailor et al. used a modified AAV6 capsid, termed AAV6.2FF, to deliver the hACE2 gene to the lungs of mice via intranasal administration and render wild-type mice susceptible to SARS-CoV-2 infection [53]. BALB/c and C57BL/6 mice of different ages and sexes were administered 1 × 10^11^ vg of AAV6.2FF-hACE2 or AAV6.2FF-Luciferase via a modified intranasal administration method [84]. Ten days after AAV transduction, the mice were inoculated with 10^5^ TCID_50_ of SARS-CoV-2 and were sacrificed at 2, 4, or 28 days post-infection. Mice transduced with AAV-hACE2 yielded high SARS-CoV-2 titers in the lungs and nasal turbinates, developed an IgM and IgG antibody response, and showed a modulation of the cytokine profiles in the lung and nasal turbinate tissues, further suggesting the development of the successful mouse model. The authors found that while age and sex did not affect SARS-CoV-2 infection characteristics, there were significant strain-related differences between the BALB/c and C57BL/6 mice.

Gary and colleagues were the first to use a model of AAV-vectorized expression of hACE2 to test a vaccine against SARS-CoV-2 [46]. In this case, BALB/c mice were prime-boost vaccinated intramuscularly with plasmid DNA encoding the SARS-CoV-2 spike glycoprotein (INO-4800) or a control gene. After vaccination, and 14 days prior to challenge with SARS-CoV-2, the mice were intranasally administered 1 × 10^11^ vg of a lung-tropic AAV6.2FF vector expressing hACE2 from the composite CASI promoter. Assessment of the viral load in the lungs of vaccinated mice four days post-challenge revealed that a single dose of vaccine resulted in 50% of animals with no detectable virus in the lungs, whereas a prime-boost immunization was completely protective. This study highlights the utility of the AAV-hACE2 mouse model for preclinical evaluation of novel SARS-CoV-2 vaccines.

Sun et al. employed a dual-AAV-serotype, multi-route transduction approach to promote the widespread expression of hACE2 for rapid development of a mouse model for SARS-CoV-2 [47]. C57BL/6 mice were transduced with 3 × 10^11^ vg of AAV6-CMV-hACE2 intratracheally (I.T.) and 1 × 10^12^ vg of AAV9-CMV-hACE2 intraperitoneally (I.P.) to ensure systemic expression of hACE2 and render the mice susceptible to SARS-CoV-2. Robust expression of hACE2 was detected in the lung, heart, and liver as early as 1 week post-transduction and persisted systemically for at least 28 weeks. AAV-mediated hACE2 expression rendered wild-type mice susceptible to SARS-CoV-2 infection, leading to high levels of virus replication in the lung and other extrapulmonary tissues, moderate to severe pathology in the lungs with features similar to those observed in patients with severe COVID-19, and significant weight loss. Moreover, this novel and adaptable approach for the rapid development of a mouse model for an emerging infectious disease was achievable within one month.

Using the same combinatorial approach as described above [47], Wang et al. evaluated the efficacy of a novel RBD protein/peptide vaccine. Vaccinated mice were challenged two weeks post-dual-vector administration (AAV6-hACE2 I.T. and AAV9-hACE2 I.P.) [83]. The high-dose vaccine group displayed significantly lower viral loads in the lung and a significant reduction in lung pathology compared with the mock-transduced mice. The success of this preclinical mouse model led to the subsequent evaluation of this promising vaccine candidate in a non-human primate model, which also demonstrated dose-dependent protection.

The AAV-hACE2 model has also been used to evaluate the efficacy of antiviral agents against SARS-CoV-2. Li et al. tested the efficacy of the remdesivir metabolite GS-441524 in mice intratracheally administered 5 × 10^11^ vg of AAV9-hACE2 and challenged 30 days later with SARS-CoV-2. Mice administered GS-441524 intraperitonially one day prior to infection and for eight days after treatment showed significantly reduced SARS-CoV-2 replication in vivo [49].

Several other groups have published studies involving the AAV-vectorized expression of hACE2 [47,48,50,51,54]. For a summary of the AAV-hACE2 models used to study SARS-CoV-2 infection, pathogenesis, and various intervention strategies, see Table 2.

#### 4.2.2. Adenovirus-Vectorized Expression of hACE2

Sun et al. described one of the first uses of adenovirus-vectored expression of hACE2 to sensitize mice to SARS-CoV-2 infection [55]. In this study, several strains of WT (C57BL/6, BALB/c) and KO mice (IFNAR^−/−^, STAT1^−/−^, IFN-γ^−/−^) were intranasally administered 2.5 × 10^8^ PFU of replication-defective Ad5-hACE2 and, five days later, challenged with SARS-CoV-2. Weight loss and high-titer virus replication were detected in the Ad5-hACE2-sensitized mice in addition to severe pulmonary pathology indicative of pneumonia. The contribution of type I and type II IFN signaling to COVID-19 lung disease was investigated in Ad5-hACE2-transduced IFNAR^−/−^ and IFN-γ^−/−^ mice, respectively. While the absence of type I IFN signaling delayed virus clearance and diminished inflammation, the lack of type II IFN signaling had no apparent effect on clinical signs, virus replication, or pathological changes in the lung. SARS-CoV-2 infection of Ad5-hACE2-sensitized STAT1^−/−^ mice, on the other hand, resulted in greater weight loss, enhanced immune cell infiltration into the lungs, and delayed virus clearance. Another advantage of the vectorized hACE2 approach in mice is the easy access to reagents that can be used to deplete specific subsets of immune cells to evaluate their contribution to viral pathogenesis and vaccine efficacy. Depletion of CD4+ and CD8+ T cells individually or in combination from Ad5-hACE2-sensitized mice revealed that a dependence on both cell types for optimal SARS-CoV-2 clearance was required. Passive transfer of serum from vaccinated mice revealed the requirement for neutralizing antibodies in SARS-CoV-2 clearance from infected lungs. Finally, the authors interrogated the efficacy of SARS-CoV-2 therapeutics including remdesivir and convalescent sera from human patients who had recovered from SARS-CoV-2 infection using the Ad5-hACE2-transduced mouse model.

In a coordinated publication with Sun et al. [55], Hassan et al. [56] described similar findings using the Ad5-hACE2-sensitized mouse model. In this instance, an anti-IFNAR1 monoclonal antibody was used to transiently inhibit type I IFN signaling in Ad5-hACE2-transduced BALB/c mice, again highlighting the rapid and flexible nature of this alternative mouse model for SARS-CoV-2 infection. In a follow-up publication from the same group, the Ad5-hACE2-sensitized mouse model was used to evaluate the efficacy of a VSV-vectored vaccine expressing the SARS-CoV-2 spike protein. Intraperitoneal administration of the VSV-eGFP-SARS-CoV-2 vaccine induced high-titer neutralizing antibodies and protected against lung infection, inflammation, and pneumonia, highlighting the important role that vaccine-induced neutralizing antibodies play in mediating protection [57].

Following these initial publications, several groups reported using the Ad5-hACE2-sensitized mouse model for detailed pathogenesis studies [58,59,60,62], testing vaccine candidates, and evaluating the efficacy of various therapeutic agents, including receptor-binding domain (RBD)-blocking antibodies [61]. The details of these studies are summarized in Table 3.

### 4.3. Common Cold Coronaviruses (CCCoVs)

In addition to the newly emerged coronaviruses that cause respiratory infections in humans, there are four common cold coronaviruses (CCCoVs), namely, HCoV-229E, HCoV-OC43, HCoV-NL63, and HCoV-HKU1, that have been endemic in the human population for a long period of time and are typically associated with mild upper respiratory infections [85]. As with other human coronaviruses, mice are not naturally susceptible to these viral infections due to the absence of the appropriate viral receptors. As was done for MERS-CoV and SARS-CoV-2, Lui et al. established mouse models for HCoV-229E and HCoV-NL63 by introducing their respective receptors, human aminopeptidase N (hAPN) and human angiotensin-converting enzyme 2 (hACE2), via intranasal instillation of adenoviral vectors [86]. These models proved to be valuable for studying T-cell responses and identifying immunodominant epitopes as well as for testing Venezuelan equine encephalitis replicon particle vaccine candidates expressing spike proteins of 229E and NL63, both of which accelerated viral clearance in these mice. Finally, Ad5-hAPN- and Ad5-hACE2-sensitized mice infected with 229E or NL63 exhibited partial protection against SARS-CoV-2 infection, which, through immune cell depletion studies, was found to be mediated in part by T cells.

### 4.4. Hepatitis B Virus

Chronic hepatitis B virus (HBV) infection represents a significant public health burden. Although effective vaccines became available in 1998, there remain 250 million individuals suffering from chronic HBV worldwide [87]. Due to the asymptomatic nature of HBV, it has been estimated by the Centers for Disease Control (CDC) that one-third or fewer of all HBV-infected individuals in the USA are aware of their infection [88]. Notwithstanding HBV’s asymptomatic nature, it remains an important cause of hepatocellular carcinoma (HCC) worldwide [89]. Attempts to control HBV infections are complicated by the fact that there are currently 10 genotypes with varying distributions throughout the world [90]. Because of this, research efforts are ongoing to find an effective cure for HBV. Currently, mice are limited in their utility as a model for HBV since they do not fully replicate the spread of HBV infection within the mouse liver and they exhibit minimal or mild signs of hepatitis [91]. Mice are not naturally susceptible to HBV infection, ostensibly due to the lack of functional HBV receptors on mouse hepatocytes. Since no other rodent laboratory models are readily available, there are limited options for preclinical therapeutic testing against this virus. While HBV transgenic mice with a chromosomally integrated viral genome produce infectious HBV, these mice are a poor model due to their development of immune tolerance to viral antigens and thus lack of inflammatory liver disease [92]. In 2011, the development of a novel HBV mouse model using AAV was reported. Unlike the previously described approaches for human coronaviruses, which vectorized the viral receptor, Huang et al. vectorized the HBV genome in an effort to bypass the attachment and entry step, allowing for the initiation of the HBV replication cycle in a mouse model [93]. To satisfy safety requirements, the authors used a trans-splicing approach, whereby the HBV genome was split into two AAV vectors, each containing approximately half of the HBV genome, flanked with splice donor or acceptor sequences [94]. It was hypothesized that head-to-tail intermolecular concatamerization of the co-transduced vectors would result in functional HBV genomic and messenger RNAs and reconstitution of the HBV genome. Co-transduction of BALB/c mice intravenously with trans-splicing AAV vectors resulted in the sustained production of HBV DNA and virions in the serum in a dose-dependent manner. Production of HBV virions and corresponding proteins was further confirmed in the liver and blood in all four different strains of immunocompetent mice transduced with AAV/HBV, with antigen production and antibody profiles similar to those observed in chronic HBV patients. Importantly, 12–16 months post-AAV/HBV administration, all 12 AAV/HBV-transduced mice developed macroscopically visible liver tumor nodules. Ten of the twelve tumors were characterized as having typical HCC features. This AAV/HBV-induced HCC model presents a valuable platform for investigating the pathogenic mechanisms underlying HBV-associated HCC and for facilitating the development of therapeutic interventions targeting HCC.

### 4.5. Hantavirus

Hantaviruses are a family of viruses (*Hantaviridae*) that are found worldwide and that cause the human diseases hemorrhagic fever with renal syndrome (HFRS) and hantavirus cardiopulmonary syndrome (HCPS) [95]. Hantaviruses are rodent-borne viruses, and reservoir hosts are infected throughout their lifetime, often with little to no disease development [96]. Hantavirus infection become well known during the Korean War from 1950 to 1953. There are about 40 species of hantavirus that have been identified, 22 of which are pathogenic to humans, and all have rodents as reservoirs [97,98]. Old World hantaviruses found in Europe and Asia cause HFRS and have a mortality rate of approximately 1–15%. Old World viruses include Puumala virus, Dobrava-Belgrade virus, and Hantaan virus. New World hantaviruses exist in the Americas and cause HCPS. HCPS has a higher mortality rate, ranging from 25% to 40%, depending on the causative virus. New World viruses include Sin Nombre virus (SNV), Choclo virus, and Andes virus (ANDV). Although there is a vaccine, Hantavax, approved for use in China and Korea for the Hantaan virus [99], there are currently no FDA-approved vaccines nor antivirals for hantaviruses, and treatments and preventative measures for HCPS are sorely needed. Currently, the only small-animal model of HCPS is the Andes virus model in Syrian hamsters [100,101]. In terms of NHPs, cynomolgus monkeys are susceptible to infection with wild-type Puumala virus, an Old World hantavirus, and Rhesus macaques are susceptible to Sin Nombre virus infection, resulting in HCPS development [102,103,104]. Wild-type laboratory mice are not susceptible to New World hantavirus infection; however, there are Hantaan virus infection models in mice, including immunocompromised or newborn mice or mouse-adapted virus passaged in newborn mouse brains [105,106,107,108]. Historically, experiments aimed at identifying receptors for hantavirus entry identified α_v_β_3_ integrins, which were shown to be critical for the entry of pathogenic hantaviruses, while α_v_β_1_ integrins facilitate the entry of non-pathogenic hantaviruses. However, recent studies have identified protocadherin-1 (PCDH1) as a critical host factor for New World hantaviruses [109]. Protocadherin-1 is a cadherin superfamily member, and although its precise role remains to be elucidated, it represents a new antiviral target and a possible candidate for vectorized receptor expression [110]. To date, no studies evaluating vectorized expression of PCDH1 as a method to develop a hantavirus infection model in mice have been published. However, it has been shown recently that introducing human PCDH1 into primary murine lung microvascular endothelial cells (MLMECs) increased infection of vesicular stomatitis virus (VSV) pseudotyped with the GPC from the Sin Nombre virus (VSV-SNV-Gn/Gc), suggesting that a molecular mismatch between Sin Nombre GPC and murine PCDH1 might be partially accountable for the entry restriction observed in murine cells [110]. Indeed, a single amino acid residue in the extracellular domain of PCDH1 was found to influence the host species specificity of SNV glycoprotein–PCDH1 interaction and cell entry [110]. These findings suggest that using AAV or AdV to express the human PCDH1 in mice might facilitate the generation of a mouse model for New World Sin Nombre and Andes hantaviruses, and it is a line of research that our laboratory is actively pursuing. Given the high rates of mortality caused by hantaviruses, their ability to spread via aerosol transmission, and the lack of existing treatments, it is of high importance that reliable and easily accessible mouse models supporting hantavirus infection are developed to facilitate rapid and efficacious hantavirus vaccines [111].

## 5. Conclusions

In conclusion, the establishment of animal models for researching viral diseases holds immense significance, enabling the assessment of vaccine and therapeutic safety and efficacy before advancing to clinical trials. The lack of suitable animal models for certain viruses imposes considerable challenges to researchers looking to pursue this area of research, since the cost of developing, producing, and breeding transgenic animal models is high. Immediately following the development of a suitable animal model is the costly and lengthy development process of producing a therapeutic or prophylactic. Currently, AAV and AdV vectors are the gold standard for in vivo gene delivery and provide researchers with a tool to render mice, and potentially other animal species, susceptible to infection with viruses that otherwise lack natural hosts or readily available models. Notably, viruses like SARS-CoV-2 and MERS-CoV, which do not naturally infect mice, have been made permissive to mice using the vectored expression of hACE2 or hDPP4, respectively, thereby facilitating the evaluation of various prophylactic and therapeutic treatments. Although this approach has not yet been investigated for hantaviruses, it is highly likely that vectored expression of the human PCDH1 receptor will increase the permissiveness of mice to this virus. Ideally, other high-consequence pathogens can be evaluated in mouse models using this vectorized receptor expression approach, thereby allowing for the rapid development of versatile mouse models of infection using any strain of mouse, including knockout mice (e.g., IFN receptor knockout mice), or animal species that are transducible by AAV or AdV vectors.

While there are reports in the literature of direct comparisons between SARS-CoV-2 infection in the K18-hACE2 transgenic mouse model and the Ad5-hACE2 mouse model, as well as different serotypes of AAV-hACE2, we are not aware of any publications that have undertaken a head-to-head comparison of AAV- and AdV-vectored expression of hACE2, or any other viral entry receptor, in a challenge model. Nonetheless, this would be an important contribution to the field.

## Figures and Tables

**Table 1:** Summary of the advantages and disadvantages of viral-vectored receptor expression for developing mouse models of viral infection.

Advantages	Limitations
Rapid model development: simply need to synthesize and clone the receptor gene and produce viral vector.	Requires a priori knowledge of the receptor molecule(s) that the virus uses to enter cells.
Ability to use any strain of commercially available laboratory mice, including aged mice and transgenic mice, e.g., IFNR^−/−^, STAT1^−/−^, Sting^−/−^, which can be purchased quickly and easily in large numbers if necessary. There is also the potential to use other animal species, including Syrian hamsters, ferrets, etc. However, the use of other species may be contraindicated and/or pose additional challenges beyond what is presented for mice, including the need for much larger doses of the vector and the possibility of an immune response to the transgene, leading to rapid clearance of vector-transduced cells.	This approach will not be able to overcome intracellular blocks to virus replication unless there are readily available transgenic mice that already have this block to virus replication knocked out. However, this would also be a limitation in transgenic models as well.
Ability to perform sophisticated immunological studies due to the plethora of reagents readily available for studies involving mice.	Targeting specific organs, such as the lungs, liver, or brain, is better suited for vectored receptor expression strategies compared with those that necessitate widespread or blood cell receptor expression.
Can use authentic/natural virus isolates without having to go through the lengthy process of mouse or other species adaptation.	Potential for mouse-to-mouse variations in transgene expression, as well as variability in transgene expression between tissues.
Use of authentic virus in challenge studies allows for more accurate testing of antibody therapies that target the receptor-binding domain (RBD) of the virus	Mild bronchial inflammation is associated with AdV delivery.
Ability to express more than one protein involved in the virus life cycle, for example, hACE2 and TMPRSS2 in the case of SARS-CoV-2.	The potential for mouse-to-mouse variation in transduction efficiency could lead to transgene expression differences.
Ability to administer the vector via any route of administration and with inducible promoters for controlled receptor expression.	
Possible to evaluate co-infection in a mouse model by using vectored delivery of two virus receptors, e.g., hACE2 and hDPP4 to study SARS-CoV-2 and MERS-CoV co-infection.	

**Table 2:** Summary of publications that have employed the AAV-vectorized expression of the hACE2 mouse model of SARS-CoV-2 infection.

AAV Serotype	Promoter	Dose (vg), Route of Administration, and Time Between AAV-hACE2 Transduction and SARS-CoV-2 Challenge	Strain of Mice	Interventions Tested	Reference
AAV9	CMV	1 × 10^11^ vg IT and challenged 2 weeks later	Male and female C57BL/6J (B6J), IFNAR^−/−^ IRF3/7 double knockout	N/A	Israelow et al., 2020 [45]
AAV6.2FF	CASI	1 × 10^11^ vg IN and challenged 2 weeks later	Male and female BALB/c	DNA vaccine	Gary et al., 2021 [46]
AAV6 and AAV9	CMV	3 × 10^11^ vg IT (AAV6) and 1 × 10^12^ vg IP (AAV9), challenged 2 weeks later	C57BL/6J, BALB/c	Cocktail of chimeric anti-SARS-CoV-2 spike RBD monoclonal antibodies	Sun et al., 2021 [47]
Not provided	Not provided	1.6 × 10^11^ vg IN and challenged 2 weeks later	C57BL/6J Nlrp3^−/−^	NLRP3-specific inhibitor MCC950	Zeng et al., 2022 [48]
AAV9	Not provided	5 × 10^11^ vg IT and challenged 30 days later	BALB/c	Remdesivir metabolite GS-441524	Li et al., 2022 [49]
AAV8	CAG	4 × 10^11^ vg IT and 6 h later with 4 × 10^11^ vg IN; challenged 1 week later	C57BL/6, TLR7^−/−^, NOD2^−/−^, IFNAR^−/−^	N/A	Yang et al., 2022 [50]
AAV6, AAV9, AAVDJ	Not provided	2–6 × 10^10^ vg IN and challenged 10 days later	BALB/c	N/A	Glazkova et al., 2022 [51]
AAV6 and AAV9	CMV	3 × 10^11^ vg IT (AAV6) and 1 × 10^12^ vg IP (AAV9) and challenged 2 weeks later	BALB/c	Subunit vaccine UB-612	Wang et al., 2022 [52]
AAV6.2FF	CASI	1 × 10^11^ vg IN and challenged 10 days later	Male, female, old, and young C57BL/6 and BALB/c	N/A	Tailor et al., 2022 [53]
AAV6	CMV enhancer/beta-actin (CB) promoter	3 × 10^11^ vg IT; the interval between AAV and SARS-CoV-2 challenge was not provided	C57BL/6	Anti-PD-L1 antibody	Huang et al., 2023 [54]

vg = vector genome, IN = intranasal, IT = intratracheal, IP = intraperitoneal, N/A = not applicable.

**Table 3:** Summary of publications that have employed the adenovirus-vectorized expression of the hACE2 mouse model of SARS-CoV-2 infection.

AdV Serotype	Promoter	Dose (vg), Route of Administration, and Time Between AAV-hACE2 Transduction and SARS-CoV-2 Challenge	Strain of Mice	Interventions Tested	Reference
Ad5	CMV	2.5 × 10^8^ PFU IN and challenged 5 days later	BALB/c, C57BL/6, IFNAR^−/−^, STAT1^−/−^	Venezuelan equine encephalitis replicon particles (VRPs) expressing the SARS-CoV-2 spike (VRP-S), transmembrane (VRP-M), nucleocapsid (VRP-N), and envelope (VRP-E) proteins, human convalescent plasma, two antiviral therapies (poly I:C and remdesivir)	Sun et al., 2020 [55]
Ad5	CMV	2.5 × 10^8^ PFU IN and challenged 5 days later	BALB/c	Anti-SARS-CoV-2 mAb 1B07	Hassan et al., 2020 [56]
Ad5	CMV	2.5 × 10^8^ PFU IN and challenged 5 days later	BALB/c	VSV-eGFP-SARS-CoV-2 vaccine	Case et al., 2020 [57]
Ad5	CMV	7.5 × 10^7^, 1 × 10^8^, or 2.5 × 10^8^ PFU IN and challenged 5 days later	BALB/c C57BL/6	N/A	Wong et al., 2020 [58]
Ad5	CMV/K18	2.5 × 10^8^ PFU IN and challenged 5 days later	BALB/c C57BL/6	N/A	Rathnasinghe et al., 2020 [59]
Ad5	Not provided	1.5 × 10^9^ PFU oropharyngeal and challenged 5 days later	C57BL/6	N/A	Han et al., 2021 [60]
Ad5	Not provided	2.5 × 10^8^ PFU IN; interval between transduction and challenge not specified	BALB/c	SARS-CoV-2 neutralizing antibody P2C-1F11	Ge et al., 2021 [61]
Ad5	Not provided	2.5 × 10^8^ PFU IN and challenged 5 days later	BALB/c C57BL/6	N/A	Zhuang et al., 2021 [62]
Ad5	Not provided	4 × 10^8^ TCID50 IN and challenged 5 days later	IFNAR^−/−^	SARS-CoV-2 neutralizing antibody PR1077	Fu et al., 2021 [63]
Ad5	Not provided	1.5 × 10^9^ PFU oropharyngeal and challenged 5 days later	C57BL/6	N/A	Liu et al., 2021 [64]
Ad5	Not provided	2.5 × 10^8^ PFU IN and challenged 5 days later	BALB/c	Serine protease inhibitors (camostat mesylate and nafamostat mesylate)	Li et al., 2021 [65]
Ad5	CMV	2.5 × 10^8^ PFU IN and challenged 5 days later	BALB/c	Anti-SARS-CoV-2 mAb NC0321 expressed from IN administered rSIV.F/HN lentiviral vector	Du et al., 2022 [66]
Ad5	CMV	2.5 × 10^8^ PFU IN and challenged 5 days later	C57BL/6	N/A	Yang et al., 2022 [67]
Ad5	CMV	2.5 × 10^8^ PFU IN and challenged 5 days later	C57BL/6	IgY-RBD antibody	El-Kafrawy et al., 2022 [68]
Ad5	CMV	2.5 × 10^8^ PFU IN and challenged 5 days later	IFNAR^−/−^, STAT1^−/−^	Investigated whether pre-existing immunity to seasonal CoV spikes could have a protective effect against SARS-CoV-2	Liu et al., 2023 [69]

vg = vector genome, IN = intranasal, IT = intratracheal, IP = intraperitoneal, N/A = not applicable.

## Data Availability

No new data were created.
